# Practice patterns in reporting and documentation of Charles Bonnet syndrome: a retrospective review following COVID-19

**DOI:** 10.1177/25158414241232285

**Published:** 2024-03-27

**Authors:** Dalia Abdulhussein, Lee Jones, Sri Harsha Dintakurti, Mariya Moosajee

**Affiliations:** NIHR Biomedical Research Centre at Moorfields Eye Hospital NHS Foundation Trust, London, UK; UCL Institute of Ophthalmology, London, UK; Imperial College London, London, UK; UCL Institute of Ophthalmology, 11-43 Bath Street, London EC1V 9EL, UK; NIHR Biomedical Research Centre at Moorfields, Eye Hospital NHS Foundation Trust, London, UK; Department of Ophthalmology, Great Ormond Street Hospital for Children NHS Trust, London, UK; The Francis Crick Institute, London, UK

**Keywords:** Charles Bonnet syndrome, visual hallucinations

## Abstract

**Background:**

Charles Bonnet syndrome (CBS) is characterized by visual hallucinations occurring in people with visual impairment. CBS can negatively impact psychological well-being, and the COVID-19 pandemic period was associated with an exacerbation of symptoms.

**Objectives:**

To compare clinical practice patterns and reporting of CBS at a tertiary eye care center between an interval prior to the COVID-19 pandemic and an interval during the pandemic.

**Design:**

Retrospective chart review.

**Methods:**

A search of electronic medical records for all suspected CBS cases was conducted between 1 March 2019 and 29 February 2020 (prior pandemic interval) and between 1 September 2020 and 29 August 2021 (peri-pandemic interval). Data retrieved from records included patient demographics, visual acuity at the time of CBS onset, type of hallucinations, reporting healthcare professional, management strategies and patient-reported impact of hallucinations.

**Results:**

In total, 223 appointments referred to CBS in 156 patients at the prior interval, while 239 appointments referred to CBS in 155 patients at the peri-pandemic interval, representing 0.07% and 0.09% of all hospital attendance, respectively. Clinical subspecialty where CBS was most commonly reported was medical retina, and a greater proportion of patients at both time intervals were female. Types of hallucinations, management strategies and patient-reported impact were seldom reported, although documentation improved at the latter interval.

**Conclusion:**

Practice patterns and patient characteristics were similar between the two intervals; however, subtle differences suggest a growing awareness of CBS. Targeted interventions in high-burden clinical subspecialties may encourage reporting and improve documentation of CBS.

## Introduction

Charles Bonnet syndrome (CBS) describes the phenomenon of vivid visual hallucinations secondary to sight loss, occurring without voluntary control. These hallucinations may occur independently as a consequence of visual impairment, with or without additional neuropsychiatric factors.^1^ Typical phenomenology ranges from unformed shapes and irregular patterns (e.g. tesselopsia and dendropsia) to more complex formed images comprising of animals, people and life-like scenes.^2^

Although the precise pathogenesis is unknown, evidence suggests CBS is a consequence of deafferentation, whereby diminished sensory input from the eyes results in spontaneous hyper-excitability of the visual cortex, causing hallucinations.^3,4^ Prevalence estimates for CBS vary depending on specific patient populations under investigation.^2^ A recent meta-analysis indicated the pooled prevalence of CBS among low vision patients (better eye Snellen 6/18 or worse) aged over 40 years to be approximately 20%, suggesting a global estimate of over 47 million patients.^5^ These estimates are significant, given a common presumption that CBS is rarely seen in eye clinics.

Variation in the reported prevalence may be explained by wide and/or ambiguous diagnostic criteria for the condition. For example, CBS is sometimes used to refer to complex hallucinations only.^6^ Other challenges include an underreporting of the condition due to insufficient awareness among healthcare professionals, self-perceived irrelevance of visual hallucinations to eye health and that patients retain insight that their hallucinations are not real and, therefore, seldom voluntarily disclose their symptoms due to concerns that hallucinations are indicative of psychiatric illness.^7–9^

Although onset of CBS is attributed to deafferentation, environmental factors may influence hallucination phenomenology. Social isolation (i.e. a person’s separation from significant others, groups, activities and situations) may amplify the negative effects of CBS. For example, several studies associate living alone with increased reporting of CBS,^10–12^ however, these findings are not always replicated.^13,14^ Moreover, loneliness and isolation during the COVID-19 pandemic have been identified as a potential trigger for changes in hallucination phenomenology.^15^

Given the challenges associated with effective CBS case finding and the potential disease-modifying effects of the COVID-19 pandemic, specific analysis of the recording of CBS during the pandemic can shed light onto clinical practice patterns and reporting behaviours. The aim of this study was to compare reporting of CBS prior to the COVID-19 pandemic with a period at the height of the pandemic, with specific focus on patient characteristics, management strategies and patient-reported impact.

## Materials and methods

**Figure 1.**
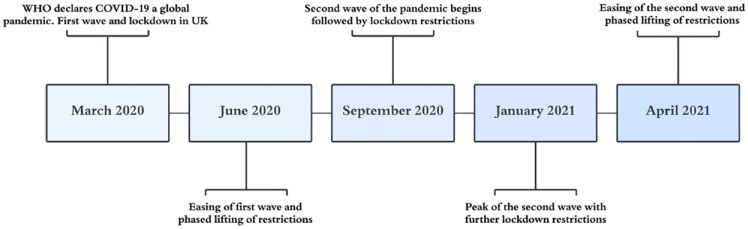
Timeline of the COVID-19 pandemic in the United Kingdom.

Patients with suspected CBS were flagged using terms ‘Charles Bonnet’ and ‘visual hallucinations’ in a search engine of electronic patient records at Moorfields Eye Hospital (MEH) NHS Foundation Trust with an age limit of 16 years and older. We also ensured we captured possible data by using wider search terms, including ‘CBS’, ‘Bonet’, ‘Bonnet’ and ‘hallucination’. Date restrictions were applied to retrieve patients attending clinics between 1 March 2019 and 29 February 2020 (i.e. prior to the COVID-19 pandemic). We subsequently retrieved patients seen at the height of the pandemic between 1 September 2020 and 29 August 2021. A brief outline of the COVID-19 pandemic in the United Kingdom is summarized in Figure 1. OpenEyes (Across Health, Ghent, Belgium) electronic database was used to retrieve data on demographics and descriptive clinical features. The following were retrieved from records: sex, ethnicity, age, ocular diagnosis, co-morbidities and best-corrected visual acuity (BCVA) at time of diagnosis. BCVA is reported using LogMAR. For this study, very low vision recorded as counting fingers was valued at 1.9 LogMAR, hand motion was 2.3 LogMAR, perception of light was 2.7 LogMAR and no perception of light was valued at 3.0.^16,17^ Where available, descriptors of the visual hallucinations experiences, impact on patient, reporting healthcare professional and subsequent management were included. In addition, outpatient attendances at the hospital across all clinical services, excluding paediatrics, were analysed to establish proportions of cases reported between the two time periods. Both face-to-face and telemedicine consultations were captured in this search.

### Data analysis

Statistical analysis was performed using SPSS version 27.0 (SPSS Inc., Chicago, IL, USA). One sample binomial test was used to analyse sex data to observe whether proportions differed from expected results. Differences between the two time intervals were compared using paired *t* tests for mean scores and Pearson’s χ^2^ for categorical data.

## Results

### Overall number of outpatient appointments

Data was obtained for the total number of outpatient appointments, both face-to-face and telemedicine consultations, at MEH between the two time periods. Between 1 March 2019 and 29 February 2020, there were a total of 314,890 outpatient appointments attended across all ophthalmic subspecialties (including support services and research and development). During this period, 223 appointments involved reporting an established or a working diagnosis of CBS, representing 0.07% of all outpatient appointments within the same timeframe. Between 1 September 2020 and 29 August 2021 a total of 259,313 outpatient appointments were attended across all specialties. During this period 239 appointments involved reporting of an established or a working diagnosis of CBS, representing 0.09% of all appointments.

### Patient characteristics

Between 1 March 2019 and 29 February 2020, 223 appointments referred to CBS from a total of 156 patients. Of these patients, 120 (76.9%) had a new diagnosis with no prior reporting in any previous clinic letters. Between 1 September 2020 and 29 August 2021, 239 appointments referred to CBS from a total of 155 patients. Of these, nine were also captured in the initial search during the March 2019 to February 2020 period. A total of 105 patients (67.7%) had a new diagnosis of CBS during this period with no prior reporting in any previous clinic letters. There was no statistically significant difference in the number of new diagnoses between the two intervals (76.9% *versus* 67.7%; *p* = 0.07, Pearson’s χ^2^).

**Figure 2.**
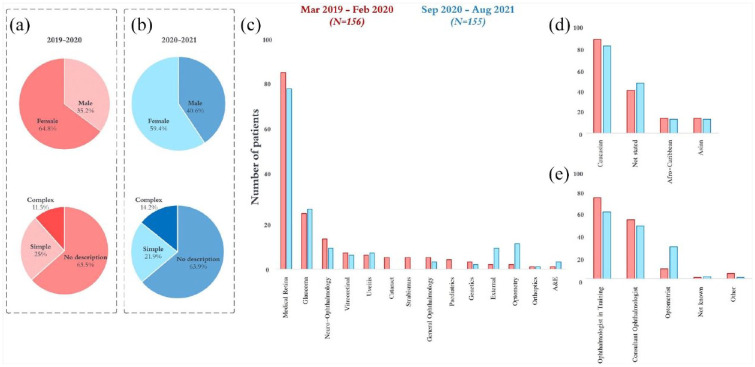
Results extracted from patient records in the pre-pandemic interval are shown in red (*N* = 156) and the peri-pandemic interval are shown in blue (*N* = 155). Graphs (a) and (b) show sex distributions and descriptors of hallucinations between intervals. Graph (c) shows numbers identified based on clinical subspeciality. Graph (d) shows ethnicity. Graph (e) shows the reporting healthcare professional.

Characteristics of patients identified during each period are summarized in Table 1 and Figure 2. Medical retina clinics had the greatest number of records referring to CBS at both intervals, followed by glaucoma. The proportion of females with a record of CBS was greater than males at both the pre-pandemic interval (64.8% *versus* 35.2%; *p* = <0.001, one sample binomial test) and peri-pandemic interval (59.4% *versus* 40.6%; *p* = 0.03, one sample binomial test). Level of BCVA was similar at both intervals for both the better eye (*p* = 0.47, paired sample *t*-test) and worse eye (*p* = 0.22, paired sample *t*-test). A statistically significant difference in the number of patients certified as sight impaired (SI) or severely sight impaired (SSI) was observed, whereby fewer patients identified during the pandemic were registered as SI or SSI (42.9% *versus* 27.7%; *p* = 0.005, Pearson’s χ^2^).

**Table 1.** Summary characteristics of patients with CBS seen during March 2019 to February 2020 and during September 2020 to August 2021.

Characteristics	March 2019–February 2020 (*N* = 156)	September 2020–August 2021 (*N* = 155)
Age at time of letter	76 [IQR 63.5–87]	77 [IQR 64–85]
Clinic letter specialty		
A&E	1 (0.6%)	3 (1.9%)
Cataract	5 (3.2%)	0
External	2 (1.3%)	9 (5.8%)
General ophthalmology	5 (3.2%)	3 (1.9%)
Genetics	3 (1.9%)	2 (1.3%)
Glaucoma	24 (15.4%)	26 (16.8%)
Medical retina	85 (54.5%)	78 (50.3%)
Neuro-ophthalmology	13 (8.3%)	9 (5.8%)
Optometry	2 (1.3%)	11 (7.1%)
Orthoptics	1 (0.6%)	1 (0.6%)
Paediatrics	4 (2.6%)	0
Strabismus	5 (3.2%)	0
Uveitis	6 (3.8%)	7 (4.5%)
Vitreoretinal	7 (4.5%)	6 (3.9%)
Sex		
Male	55 (35.2%)	63 (40.6%)
Female	101 (64.8%)	92 (59.4%)
Ethnicity		
Afro-Caribbean	14 (9.0%)	13 (8.4%)
Caucasian	88 (56.4%)	82 (52.9%)
Asian	14 (9.0%)	13 (8.4%)
Not stated	40 (25.6%)	47 (30.3%)
Age of onset of CBS	76 [IQR 60.5–86]	77 [IQR 63–85]
BCVA at time of onset		
Better eye	[IQR 0.3–1.78]	0.8 [IQR 0.4–1.48]
Worse eye	1.8 [IQR 0.9–2.3]	1.9 [IQR 1.0–2.3]
Registered SI or SSI	67 (42.9%)	43 (27.7%)
Reporting HCP		
Consultant ophthalmologist	59 (37.8%)	53 (34.2%)
Consultant neurologist	2 (1.3%)	0
General practitioner	1 (0.6%)	0
Not known	1 (0.6%)	2 (1.3%)
Ophthalmologist in training	81 (51.9%)	67 (43.2%)
Specialist nurse	1 (0.6%)	0
Optometrist	10 (6.4%)	32 (20.6%)
Orthoptist	1 (0.6%)	1 (0.6%)

A&E, accident and emergency; BCVA, best corrected visual acuity; CBS, Charles Bonnet syndrome; HCP, healthcare professional; IQR, interquartile range; SSI, severely sight impaired; SI, sight impaired.

### Descriptors of hallucinations

Details of any specific descriptors of visual hallucinations, where they were included within patient records, were extracted. We characterized these as simple hallucinations (e.g. flashing lights, colours, simple shapes, spots), complex hallucinations (e.g. animals, peoples, complex figures) or no description. The proportions in each category at both intervals are shown in Figure 2. Descriptions of CBS were largely absent from records at both intervals.

### Management of CBS

Where recorded, we retrieved details of clinical management strategies for supporting patients with CBS. Details of management practice patterns are summarized in Table 2. At both time periods, the majority of records included no specific details on approaches to management. However, a higher proportion of records in the first time period included no details of patient management compared to the period during the pandemic (68.6% *versus* 49.0%; *p* = <0.001, Pearson’s χ^2^).

**Table 2.** Summary of the management recorded for patients with CBS during the two time periods.

Management strategy	March 2019–February 2020 (*N* = 156)	September 2020–August 2021 (*N* = 155)
Given verbal advice only	18 (11.4%)	30 (19.35%)
Given information leaflet	12 (7.7%)	17 (11%)
Directed to support services, i.e. support group, Esme’s Umbrella	6 (3.8%)	6 (3.9%)
Referred to neuro-ophthalmologist	3 (1.9%)	7 (4.5%)
Referred to psychiatrist/neurologist	4 (2.6%)	6 (3.9%)
Referred to ECLO	1 (0.6%)	9 (5.8%)
Referred to low vision services	3 (1.9%)	4 (2.6%)
Prescribed medication/intervention	1 (sleeping tablets) (0.6%) 1 (LED light) (0.6%)	0
Not described	107 (68.6%)	76 (49.0%)

CBS, Charles Bonnet syndrome; ECLO, eye clinic liaison officer.

### Impact of CBS

Few records included details on the impact of hallucinations on aspects of daily life and well-being. During March 2019 to February 2020, descriptors of the impact of CBS were reported for 28 patients compared to 19 patients between September 2020 and August 2021. Descriptors have been categorized as either ‘no impact’ or ‘negative impact’, with excerpts from clinic letters provided in Table 3.

**Table 3.** A summary of the impact of CBS on patients’ lives during the two time periods.

March 2019–February 2020 (*N* = 28)	September 2020–August 2021 (*N* = 19)
No impact on patients’ lives, *n* = 11 ‘Despite her visual hallucinations she is actually doing very well at present’ ‘Not upset by them and was relieved to hear that this is a common phenomenon’	No impact on patients’ lives, *n* = 4 ‘No effect, ignores the hallucinations’ ‘Still able to work despite hallucinations’
Negative impact on patients’ lives, *n* = 17 ‘Understands why he’s getting the images but cause him suffering’ ‘Debilitating and prevent him sleeping’	Negative impact on patients’ lives, *n* = 15 ‘Afraid of the hallucinations’ ‘Interfering with existing central vision’ ‘Impacting sleep and troubling patient daily’

## Discussion

The proportion of hospital patient records reporting symptoms of CBS was similar prior to the COVID-19 pandemic compared with a period during the height of the pandemic. Records identified during the pandemic period were more likely to include details of management strategies compared to those identified prior to the pandemic. The subspecialty where records of CBS were most commonly associated was medical retina, and a greater proportion of patients identified at both time intervals were female.

Although patients’ ophthalmological profiles were similar across both study periods, such as comparable BCVA, there were notable differences between the groups. For example, fewer patients identified during the pandemic were registered as SI/SSI compared to the pre-pandemic interval (42.9% *versus* 27.7%, respectively). Several studies have associated visual functioning with onset of CBS,^18^ and patients in this study recorded low levels of BCVA, on average, at both time intervals, including in the better-seeing eye. This finding may be explained by issues relating to registering as SI/SSI, such as uncertainty regarding eligibility, low awareness of the benefits of registration, lengthy processing measures and external pressures to reduce certification rates,^19,20^ which are likely to have been compounded during the COVID-19 pandemic.

Our results highlighted a greater likelihood for female patients to have a record of CBS compared to males. Previous studies have shown gender differences in healthcare-seeking behaviours, whereby women are more likely to report visiting their primary care provider to a greater extent than men for both physical and mental health concerns.^21^ Being male is negatively associated with willingness to seek support,^22^ and is a significant predictor of help-seeking attitudes.^23^ These attitudes are reflected in low service use, even when controlling for prevalence rates.^24^ In the context of CBS, where case identification relies primarily on opportunistic self-reporting, a greater tendency for females to have descriptions of CBS in their clinical records suggests there may be value in implementing behaviour change interventions to improve symptom reporting in men. Interventions which have shown to be successful among men share similarities such as role models to convey information, assistance with recognizing and managing symptoms, signposting services and methods which build on positive masculine traits (e.g. responsibility and strength).^25^

The number of patients with a new diagnosis of CBS was similar between the two intervals. This is interesting given there were ~55,000 fewer outpatient attendance in the period during the pandemic compared with prior to the pandemic, partly explained by fears of COVID-19 exposure being associated with a 4-fold increase in loss to follow-up in eye care centres.^26^ Although there was no statistically significant difference in the number of new diagnoses between intervals, the fact patients continued to disclose symptoms during the pandemic suggests they felt comfortable reporting their experiences despite the potential of appearing trivial given the perceived wider pressures on clinical services during the pandemic. This finding might also be due to improved history taking from healthcare professionals in relation to individual experiences of CBS. This theory is supported by the statistically significantly greater documentation of clinical management strategies reported in patient records during the latter interval. Reasons why CBS reporting and documentation were higher at the latter interval might include the recent adoption of CBS in the latest revision of the International Classification of Diseases version 11 by the World Health Organization.^27^ Furthermore, awareness of CBS continues to grow in public and professional communities through outreach campaigns, greater media profiling of the condition, training, support and establishing research funding specific to CBS.^28–30^

Across both intervals, the clinical subspeciality wherein the highest proportion of CBS cases were identified was medical retina (54.5% and 50.3%, respectively), followed by glaucoma (15.4% and 16.8%, respectively). This finding is not surprising given the greatest caseload of patients being seen within these services, and combined accounting for almost 50% of all clinical services at MEH. Common conditions within medical retina include age-related macular degeneration (AMD), diabetic retinopathy and other vascular/retinal disorders. Late-stage features of retinal disorders are often associated with damage to the foveal centralis, the area responsible for high-acuity vision densely populated with cone photoreceptors.^31^ Visual acuity is a key risk factor in the development of CBS^1,18^; thus, conditions which threaten acuity are associated with symptoms of CBS. For example, a recent systematic review and meta-analysis with data from over 4000 AMD patients found an overall prevalence of CBS of 15.8% (95% confidence interval: 11.0–21.2%).^32^

Although glaucoma was the second most common subspecialty reporting CBS cases, the proportion of patients at both time intervals was significantly lower than medical retina despite having similar caseloads. Most patients living with glaucoma will experience asymptomatic disease without observable changes to visual function until progressive damage in later stages of the disease.^33^ Indeed, a meta-analysis of the prevalence of CBS in patients with glaucoma found a lower prevalence compared to that seen in AMD. Most notably, central vision loss in glaucoma was an important risk factor for CBS.^34^

Knowledge of prevalence estimates across clinical subtypes provides important insights for clinical services and may help prioritize resources, education and training in order to maximize the efficiency of CBS case finding and the direction of support services using an approach balanced by individual patient risk. For example, in our study, there appeared to be a notable increase in the number of optometrists reporting CBS cases at the peri-pandemic interval, which may be owed to changes at an educational level, such as inclusion of CBS on optometry curriculums as well as campaigns to raise awareness of CBS among primary care providers. However, the retrospective design of the study makes it difficult to fully measure and understand these possible effects.

Clinical management of CBS at both intervals was variable and was typically not described within patient records. Clinicians often attempted to provide reassurance in clinic and guide patients to family support services and/or support group campaigns, such as Esme’s Umbrella (www.charlesbonnetsyndrome.uk). Yet, for most patients, information on clinical management and the impact of visual hallucinations on well-being were not systematically captured within patient records, and details regarding efficacy of intervention at follow-up were scarce. New developments in measuring and managing CBS may encourage better quality CBS clinical documentation. For example, a consensus framework for the management of visual hallucinations in high-burden areas, including CBS has been developed.^35^ Additionally, a novel CBS screening tool is now available to facilitate identification of CBS and to establish the patient’s need for intervention.^36^

This study has limitations. Not all CBS patients may have been identified from the selected search terms if there was no reference to the condition in the electronic records. As such, the total number of patients flagged at both intervals is likely to be an underestimation. Similarly, as patients seldom volunteer information about their symptoms due to concerns their hallucinations are indicative of neurological or cognitive problems,^37,38^ the total number of CBS cases may be higher than reported. Furthermore, this study reports findings from a single tertiary eye centre in London which limits generalizability. The retrospective design of the study differs from active case finding of CBS, which could have implications for the quality and completeness of the data. Nonetheless, this report provides useful information regarding the profile of people affected by CBS and outlines practice patterns in patient management.

In summary, the number of patients with a description of CBS in their clinical record prior to and during the COVID-19 pandemic was similar between the two time intervals. Documentation of management strategies was improved at the later time interval, which could be owed to successful awareness-raising campaigns during the pandemic period. Women were more likely than men to have symptoms of CBS documented in their records, and the greatest caseload of patients was in medical retina services. These results suggest the presence of an unmet need to encourage all those experiencing visual hallucinations to report their experiences to their healthcare team and for clinicians to ask and inform patients about CBS.

## Declarations

**Ethics approval and consent to participate:** The study adhered to the tenets set out in the Declaration of Helsinki and was approved by the London – Camden & Kings Cross Research Ethics Committee (12/LO/0141). The retrospective design of the study meant that consent was not required.

**Consent for publication:** Not applicable.

**Availability of data and materials:** Not applicable.
